# Kinetics of IgM and IgG antibodies after scrub typhus infection and the clinical implications

**DOI:** 10.1016/j.ijid.2018.03.018

**Published:** 2018-06

**Authors:** George M. Varghese, Veera Manikandan Rajagopal, Paul Trowbridge, Divya Purushothaman, Sherry Joseph Martin

**Affiliations:** Department of Infectious Diseases, Christian Medical College, Vellore 632004, Tamil Nadu, India

**Keywords:** Scrub typhus, IgM, IgG, Kinetics, Orientia tsutsugamushi, Rickettsia

## Abstract

**Objectives:**

The serological detection of IgM antibodies is the most widely used test to diagnose scrub typhus infection. However, the kinetics of IgM and IgG antibodies post-infection remain elusive, which could contribute to false positivity. The objective of this study was to document the nature of the evolution of these antibody titres after infection.

**Methods:**

Adult patients previously confirmed to have scrub typhus by IgM ELISA, positive PCR, or both, were included in this cross-sectional study. The levels of IgM and IgG antibodies in serum samples were tested using an ELISA and the distribution curve was plotted.

**Results:**

Two hundred and three patients were included in this study. Post-infection serum sampling was done between 1 month and 46 months after documented infection. IgM levels declined gradually but remained elevated above the diagnostic cut-off for up to 12 months post-infection. However, IgG levels continued to rise reaching a peak at 10 months, followed by a gradual decline over several months. In the majority of cases, the IgG levels remained above the cut-off threshold for more than 36 months.

**Conclusions:**

Clinicians need to be cautious in using a single serum sample for the detection of IgM to diagnose scrub typhus, as it remains elevated for up to 12 months after the infection, whereas the serum IgG level could be used as an indicator of past infection.

## Highlights

• The kinetics of IgM and IgG post scrub typhus infection remain elusive.
• Scrub typhus patients were followed up to study antibody kinetics.
• IgM remained above the threshold for 12 months.
• IgG reached a peak at 10 months and remained above the threshold for 36 months.
• Paired IgM samples are required for accurate diagnosis.

## Introduction

Scrub typhus is a zoonotic infection caused by the bacterium *Orientia tsutsugamushi*. It is transmitted to humans by the bite of the larval stage of trombiculid mites (Silpapojakul, 1997). With an annual incidence of about one million cases, it is an important challenge to public health in Southeast Asia (Watt and Parola, 2003). The clinical presentation may vary from a mild febrile illness to a life-threatening disseminated infection with serious complications such as acute respiratory distress syndrome, hepatitis, renal failure, meningitis, and multiple organ failure, making the diagnosis difficult (Varghese et al., 2013). An eschar at the inoculation site in patients may aid in the clinical diagnosis but is often not found (Varghese et al., 2013). The laboratory diagnosis of scrub typhus relies on serology or the detection of bacterial DNA in whole blood or eschar samples. While the indirect immunofluorescence assay (IFA) has been the reference test, this technique is expensive and often unavailable in resource-limited areas where the disease is prevalent. In addition, the interpretation of IFA results has been shown to vary greatly between operators (Phetsouvanh et al., 2013, Blacksell et al., 2007). Hence, ELISA-based estimation of IgM antibodies is now preferred (Janardhanan et al., 2014). ELISA detects IgM antibodies against the 56-kDa antigen, the major immunodominant protein located on the outer membrane of the bacteria, using a recombinant antigen (Blacksell et al., 2015). IgG antibody detection is usually used to diagnose past infection or to determine the community prevalence.

Since the diagnosis of scrub typhus is based on antibody detection, the kinetics of IgM and IgG post-infection are important; however, this has been studied minimally. Very few studies have explored the temporal pattern of IgM and IgG antibodies in scrub typhus patients (Kim et al., 2010, Chen et al., 2011). Clinical decision-making can be improved by understanding the potential persistence of detectable IgM antibody after acute infection. Therefore, the present study was performed to study the kinetics of IgM and IgG antibodies in scrub typhus patients after infection.

## Methods

Patients over 18 years of age with previously confirmed scrub typhus were included in this cross-sectional study. Scrub typhus was confirmed by positive IgM ELISA (optical density (OD) >0.8) and/or a positive PCR for *O. tsutsugamushi* at a tertiary care teaching hospital in South India between December 2011 and March 2015. Serum samples were collected and patient demographic data along with the time interval between original scrub typhus diagnosis and the time of repeat serum sample collection were documented. The study was approved by the institutional ethics committee.

ELISA for IgM and IgG antibodies was performed using the Scrub Typhus Detect kit (InBios International, Inc., Seattle, WA, USA) as per the manufacturer’s instructions. The diluted serum was transferred into 56-kDa antigen-coated microwells. After incubation, polyclonal goat anti-human IgM or IgG antibodies labelled with horseradish peroxidase enzyme and later liquid Tetramethylbenzidine (TMB) substrate were added and the reaction read at 450 nm. The kit has previously been shown to have a specificity of 98% and sensitivity of around 82–84% (Blacksell et al., 2015, Kingston et al., 2015). OD cut-off values of >0.8 for IgM and >1.8 for IgG were considered as positive for ELISA based on the testing of negative samples from asymptomatic individuals using the reference test, IFA.

## Results

**Figure 1:**
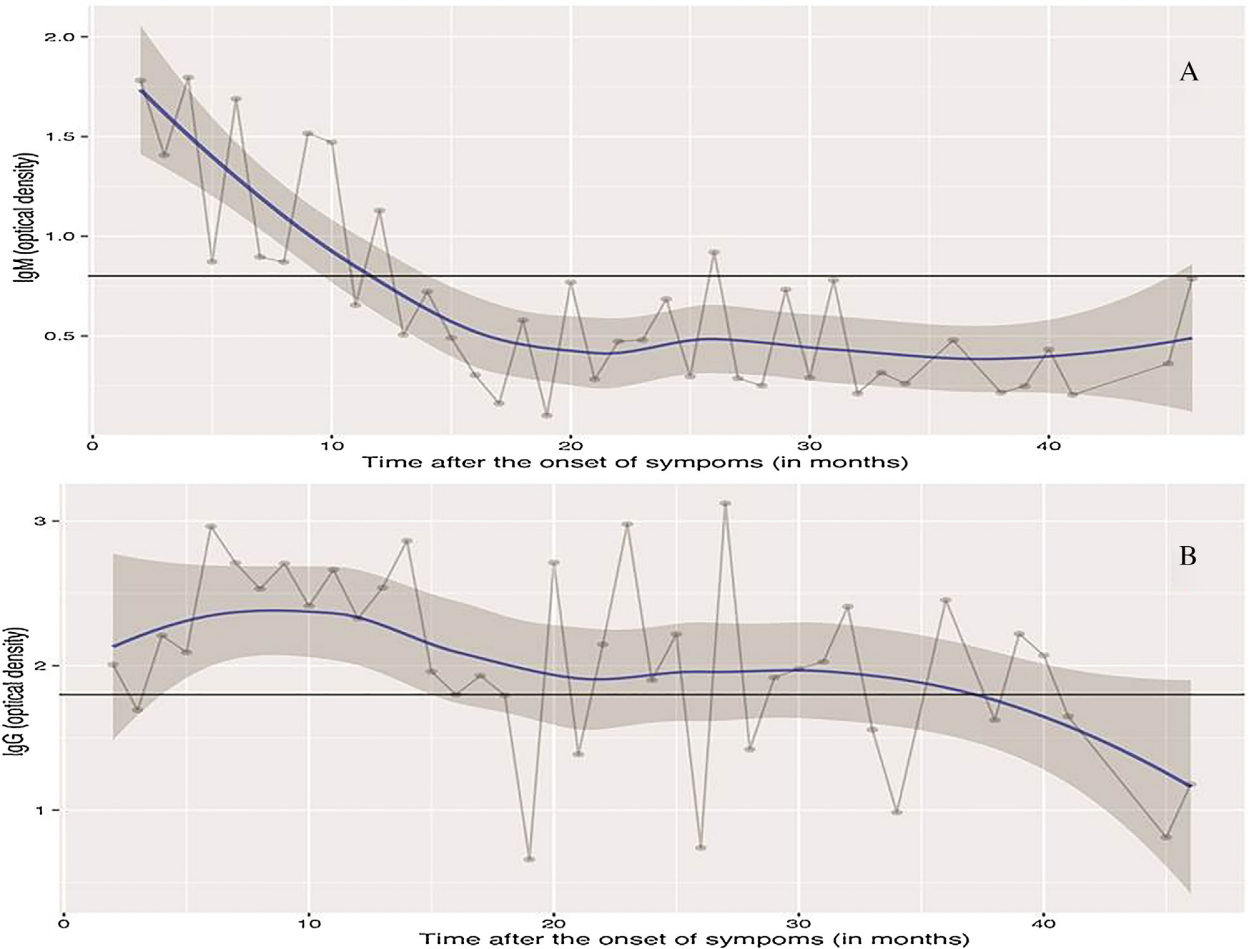
(A) Mean IgM antibody optical density plotted against time in months after confirmed scrub typhus. (B) Mean IgG antibody optical density plotted against time in months after confirmed scrub typhus.

A total of 203 patients were included in the study; 70 (35.5%) were male and 133 (65.5%) were female, and their mean age was 45.8 ± 14.6 years. All patients had a positive IgM ELISA at diagnosis, of which 80 were confirmed by PCR. All patients had a blood sampling done during the study. Among them, eight patients had two blood samples taken at different time points. The time interval between confirmation of the original infection and study sample collection ranged from 1 month to 46 months. The overall trend showed a gradual fall in mean IgM levels over time, remaining above the threshold of positivity (OD >0.8) until 12 months after infection (Figure 1A). The mean IgG levels showed a gradual rise, reaching a peak at 10 months post-infection, followed by a gradual decline, remaining above the cut-off threshold for more than 36 months (Figure 1B). The percentages of subjects with IgM and IgG levels above the threshold (IgM OD >0.8 and IgG OD >1.8) after initial infection are shown in Table 1.

**Table 1:** IgM and IgG positivity after initial infection.

Number	Time after initial infection, months	Number of patient samples	Patients with positive IgM values (OD >0.8) *n* (%)	Patients with positive IgG values (OD >1.8) *n* (%)
1	2–6	31	21 (67.74%)	20 (64.51%)
2	6–12	36	18 (50%)	29 (80.55%)
3	12–24	68	12 (17.64%)	43 (63.23%)
4	24–36	48	9 (18.75%)	26 (54.16%)
5	>36	28	2 (7.14%)	14 (50%)

OD, optical density.

## Discussion

Understanding the post-infection antibody kinetics of scrub typhus is important to guide the interpretation of IgM and IgG ELISA results. This study demonstrated that there is a gradual decline in IgM after infection and that it remains above the diagnostic threshold for about 1 year after infection. Furthermore, IgG levels peaked 10 months post-infection and remained above the cut-off level for more than 3 years post-infection.

The study results showed the persistence of IgM levels for a longer duration after infection than previously reported (Kim et al., 2010). This implies the possibility of false-positives when interpreting single serum samples for IgM antibody. Therefore, when serology is used for diagnosis, the demonstration of a rise in titres remains important, particularly if other parameters are not suggestive and the diagnosis is in doubt. PCR would greatly increase the specificity and help identify current infection in such situations (Koh et al., 2010).

IgG levels indicate previous infection and endemicity. Previous studies have reported that the peak IgG titres occur 2–3 weeks after the onset of infection (Kim et al., 2010, Chen et al., 2011). In contrast, the present study findings suggest a gradual rise in IgG levels reaching a peak at 10 months after infection and remaining above the cut-off for more than 36 months post-infection. This could also be accounted for by genetic/immunological variation between human populations or strain differences between bacteria in different endemic regions. The use of a single positive IgM ELISA as a criterion for inclusion in a proportion of patients in this study limits the potential of this study, since the results obtained indicate the possibility of false-positives in the absence of serially progressive IgM titres.
